# Favorable outcome in PLA2R positive HBV-associated membranous nephropathy

**DOI:** 10.1186/s12882-022-02871-y

**Published:** 2022-07-11

**Authors:** Ruiying Chen, Jia Wang, Qionghong Xie, Jianming Zheng, Shaojun Liu, Jun Xue, Chuanming Hao

**Affiliations:** 1 Division of Nephrology, Huashan Hospital, Fudan University, No.12, Middle Wulumuqi Road, Shanghai, 200040 China; 2 Division of Nephrology, Beijing Chao-Yang Hospital, Capital Medical University, Beijing, China; 3 Department of Infectious Diseases, Huashan Hospital, Fudan University, Shanghai, China

**Keywords:** Hepatitis B virus infection, Secondary membranous nephropathy, Phospholipase A2 receptor, Spontaneous remission

## Abstract

**Introduction:**

Over half of the patients with hepatitis B virus associated membranous nephropathy (HBV-MN) were found to be phospholipase A2 receptor (PLA2R) positive. Whether MN is really secondary to hepatitis B or just coincidence of hepatitis and PLA2R positive idiopathic MN (IMN) remains controversial.

**Methods:**

We retrospectively studied seven PLA2R positive HBV-MN patients with complete data in Huashan Hospital from 2009 to 2016 and compared them with PLA2R positive idiopathic MN patients.

**Results:**

Proteinuria and renal function of these 7 HBV-MN patients were similar to that of IMN patients. However, 5 of them were female and half showed hypocomplementemia, while in IMN group only 32.4% were female and 20% had hypocomplementemia, and the level of hematuria was 94.5/μL in HBV-MN patients and 64.9 /μL in IMN patients, though there was no statistically significant difference. Renal biopsies revealed significantly increased mesangial eletron-deposits in HBV-MN patients. All 7 patients received antiviral therapy, and one patient received immunosuppresants due to severe nephrotic syndrome with acute myocardial infarction and elevated serum creatinine. Compared with IMN group, the prevalence of remission without immunosuppressive therapy of HBV-MN patients was higher (85.7% vs. 43.7%), while the percentage of patients receiving immunosuppresants was lower (14.3% vs. 47.9%) (*P*=0.048).

**Conclusion:**

Compared with IMN patients, PLA2R positive HBV-MN patients had a more favorable prognosis after antiviral therapy, indicating a secondary form of MN. For these patients, antiviral treatment is recommended and long observation time should be provided before use of immunosuppressive treatment.

## Introduction

Hepatitis B virus (HBV) infection is a global public health problem. It’s estimated that around two billion people in the world have evidence of past or present infection with HBV, and 248 million individuals are chronic carriers [1, 2]. China is known to be one of the HBV endemic area, with an estimated 90 million (6.52%) people had chronic hepatitis B in 2016 [3]. Various extrahepatic disorders may appear in infected people, one of the commonest being HBV-associated nephropathy, which occurs in 3-5% of the patients infected [4]. Among this entity, HBV-associated membranous nephropathy (HBV-MN) is the most common renal manifestation, in which the deposition of HBeAg, HBsAg or HBcAg could be found in the subepithelial region of the glomerular basement membrane. It was first described in a 53-year-old man in 1971, but was reported more often in children later. As with idiopathic membranous nephropathy, HBV-MN presents with proteinuria which is usually in the nephrotic range. The outcome of HBV-MN is various. In children, remission is common after antiviral therapy, usually following seroconversion from HBeAg to anti-HBe. However, it’s concerning in adult patients since about 30% were reported to progress to renal failure overtime [5]. Such poor outcome has led to the development of various therapeutic approaches, resulting in the conundrum of antiviral versus immunosuppressive therapy with the risk of aggravating the viral infection.

Phospholipase A2 receptor (PLA2R) has been identified as the major antigen in idiopathic membranous nephropathy (IMN) and supposed to be involved in the pathogenesis of the disease. It could be detected in 70-80% of idiopathic MN patients, but not in patients with non-MN glomerulonephritis and healthy people [6]. In the case of secondary MN, PLA2R is hardly found in lupus nephritis but can be detected in some hepatitis B or C virus associated MN as well as sarcoidosis [7]. We have previously reported that renal PLA2R antigen could be detected in 64% of HBV-MN patients [8]. So far, sparse researches has been carried out on PLA2R positive HBV-MN patients, whether MN is really secondary to hepatitis B or just coincidence of hepatitis and PLA2R positive IMN remains uncertain. To address such question, we investigate clinicopathological features and prognosis of 7 PLA2R positive HBV-MN patients in the current study, and compare them with PLA2R associated IMN patients.

## Methods

### Study patients

Seven patients with PLA2R positive HBV-MN and seventy-one PLA2R positive IMN patients diagnosed between 2009 and 2016 in Huashan Hospital, and followed up for at least 2 years, or achieved spontaneous remission or started immunosuppressive therapy within 2 years with complete data were included. The diagnostic criteria for HBV-MN included i) the presence of a serum HBV antigen; ii) the diagnosis of membranous nephropathy, with exclusion of other kinds of secondary nephritis; and iii) the presence of HBV antigens HBsAg, HBcAg or HBeAg on renal tissue [9]. IMN was defined as biopsy-proven MN in the absence of known clinical and immunological factors causing secondary MN such as lupus and malignancy. Detailed clinical data including medical history, serological analysis and urinary test, as well as renal pathology were collected. Hypocomplementemia was defined as low C3 level (< 0.85 g/L) or C4 level (<0.10 g/L).

### Renal pathological procedure and HBsAg/HBcAg staining

A standard renal biopsy procedure including light, immunofluorescence (IF) and electron microscopy was used for renal pathological diagnosis. The extent of glomerular sclerosis, mesangial proliferation, and tubular-interstitial damage was assessed and scored semi-quantitatively. Direct IF was used for IgG, IgA, IgM, C3, C4 and C1q measurements on frozen sections of fresh tissue and scaled from 0 to 4+. Electron microscopy were used to identify dense deposits. HBV biomarkers, including HBsAg (Monoclonal Mouse Anti-HBsAg from Gene Tech, GT202429) and HBcAg (Polyclonal Rabbit Anti-HBcAg from Gene Tech, GB058629), were detected using frozen sections by indirect IF. The procedure has been described in our previous report [8].

### Detection of renal PLA2R and serum PLA2R antibodies

Renal PLA2R detection were performed on paraffin-embedded renal biopsy samples. The anti-PLA2R-Ab (Sigma, HPA012657) was diluted at 1: 500. Positive PLA2R was characterized as granular staining along the capillary loops. Negative control (secondary antibody only) was used in every case.

Circulating PLA2R-Ab was detected by indirect IF before 2016 and by ELISA after. The procedure of indirect IF has been described in our previous report [8]. Serum samples were diluted in the ratio 1: 10, 1: 100 and 1: 1,000, applied to the fixed cells expressing the full length PLA2R DNA, and incubated overnight at 4℃. A fluorescein isothiocyanate (FITC)-conjugated anti-human IgG antibody with 1: 100 dilution was used for detection of bound IgG antibodies. Transfected cells with specific cytomembrane fluorescence were considered to be positive. Commercial anti-PLA2R-Ab was used for positive control, and normal serum and secondary antibodies were used for negative control. ELISA was performed according to manufacturer's instructions (EUROIMMUN, Lübeck, Germany). Patients’ plasma was diluted 1: 100 in the phosphate buffered saline and incubated in the microplate at room temperature for 30 min. After washing, the microplate was incubated with peroxidase-conjugated anti-human IgG at room temperature for 30 min. The color was developed by adding TMB/H2O2 for 30 min and was stopped by 0.5M H2SO4. The optical density was examined at 450 nm. Samples with titer values above 20 relative light units (RU)/ml were considered positive.

PLA2R positive was defined as the presence of renal PLA2R and/or serum PLA2R antibody.

### Definition of remission and renal insufficiency

Complete remission (CR) was defined as urinary protein excretion <0.3 g/24 h, with a normal serum albumin concentration and a normal serum creatinine. Partial remission (PR) was defined as urinary protein excretion <3.5 g/24 h and a ≥ 50% reduction from peak value, accompanied by an improvement or normalization of serum albumin and stable serum creatinine. Renal insufficiency was defined as a 50% decrease in eGFR or entry of end stage renal disease

### Statistical analysis

Statistical analysis was performed using statistical software SPSS. Parametric data were presented as means±standard deviation. Nonparametric data were presented as median values with their intervals from the 25th to 75th percentile. With respect to intergroup differences of quantitative parameters, t-test was used for the comparison of serum albumin level as it was normally distributed data and Mann-Whitney U test were for other data that were not normally distributed. Differences of qualitative data were compared using Mann-Whitney U test for ordinal data like the remission rate without immunosuppresants and Fischer exact test for nominal data. All statistical analyses were two-tailed and *P* value < 0.05 was considered significant.

## Results

### Clinical characteristics

Among the 7 PLA2R positive HBV-MN patients, five were female and two were male, with a median age of 45 (31, 59) years. All 7 patients had nephrotic syndrome during disease course, with 6/7 exhibiting microscopic hematuria and 3/6 hypocomplementemia. They all presented with positive serum HBsAg, 2 patients were serum HBeAg positive and 4 patients had detectable HBV-DNA. Six patients with serum available were all positive for serum PLA2R antibody. At the time of biopsy, one patient had elevation of serum creatinine (maximum 227μmol/L) which was decreased after treatment, and another patient showed transient elevated alanine aminotransferase (maximum 343U/L) and aspartate aminotransferase (maximum 312U/L). The other 5 patients were normal for both renal and liver function (Table 1).

**Table 1:** Clinical characteristics of the 7 patients with PLA2R positive HBV-MN

**Patient**				**At the time of renal biopsy**								**Treatment**	**Follow-up**			
	Sex	age	UP (g/24)	Urine RBC (μL ^-1^)	ALB (g/L)	SCr (μmol/L)	C3 (g/L)	C4 (g/L)	HBV-DNA (IU/mL)	HBsAg /HBeAg	PLA2R-Ab titer		Time (month)	PR (month)	CR (month)	Renal insufficiency
**1**	F	30	4.97	1-2/HP	24	44	ND	ND	1.01×10^8^	+/+	1:100	ETV+ARB	61	9	23	N
**2**	F	31	7.78	351.5	20	48	0.82	0.18	7.9×10^7^	+/+	1:1000	ETV+ARB	46	14	28	N
**3**	M	33	9.48	90.8	17	82	0.55	0.13	6.45×10^3^	+/-	1:1000	ETV+ARB	45	33		N
**4**	F	45	8.09	84.8	27	47	1.53	0.35	2.8×10^3^	+/-	1:1000	ETV+ARB	97	25	52	N
**5**	F	54	3.9	155.9	26	53	1.08	0.28	UD	+/-	1:1000	ETV+ARB	24	24		N
**6**	F	59	4.00	32.3	22	50	1.15	0.30	UD	+/-	ND	LdT→ETV+ARB	58	24		N
**7**	M	69	19.65	98.2	16	121	0.69	0.31	UD	+/-	1:1000	ETV+ARB+ (FK506 + P→ CTX + P)	97	17	33	N

*F* Female, *M* Male, *UP* Urine protein, *RBC* Red blood cell, *ALB* Serum Albumin, *SCr* Serum creatinine, *HBV* Hepatitis B virus, *HBsAg* Hepatitis B virus surface antigen, *HBeAg* Hepatitis B virus e antigen, *PLA2R-Ab* Phospholipase A2 receptor antibody, *HP* High power, *UD* Undetectable, *ND* No data, *ETV* Entecavir, *LdT* Telbivudine, *P* Prednisone, *CTX* Cyclophosphamide, *PR* Partial remission, *CR* Complete remission, *ARB* Angiotensin receptor blocker, *N* No

The level of urinary protein excretion and renal function in patients with HBV-MN were similar to that of IMN patients. Five over 7 patients (71.4%) were female in HBV-MN group while only 32.4% in IMN group (*P* = 0.091), and the level of hematuria was 94.5/μL in HBV-MN patients and 64.9 /μL in IMN patients (*P* = 0.085) (Table 2).

**Table 2:** Clinical features and outcomes of PLA2R positive HBV-MN patients and idiopathic MN patients

	**HBV-MN (7)**	**Idiopathic MN (71)**	***P* value**
**Baseline clinical characteristics**			
Gender(male/female)	2/5	48/23	0.091
Age	45 (31, 59)	53 (42, 61)	0.426
eGFR (ml/min/1.73m^2^)	107.8 (102.1, 125.9)	98.3 (86.0, 109.6)	0.170
Urinary Pro (g/24h)	4.97 (2.48, 9.48)	4.74 (2.38, 8.09)	0.727
Urine RBC (/μL)	94.5 (71.7, 204.8)	64.9 (17.1, 105.3)	0.085
ALB (g/L)	24.43±5.15	26.33±6.15	0.432
Hypocomplementemia, n (%)	3/6 (50.0)	12/60 (20.0)	0.125
**Remission without IS**			0.048
NIS-Remission, n (%)	6 (85.7)	31 (43.7)	
NIS-NR, n (%)	0 (0)	6 (8.5)	
IS, n (%)	1 (14.3)	34 (47.9)	
**Renal insufficiency, n (%)**	0 (0)	8 (11.3)	1.000

*PLA2R* Phospholipase A2 receptor, *MN* Membranous nephropathy, *eGFR* estimated glomerular filtration rate, *Pro* Protein, *RBC* Red blood cell, *ALB* Serum Albumin, *HBsAg* Hepatitis B virus surface antigen, *HBeAg* Hepatitis B virus e antigen, *NIS* Non-immunosuppressive therapy, *NR* No remission, *IS* Immunosuppressive therapy

### Pathological characteristics

Pathology showed typical features of membranous nephropathy in all HBV-MN cases, including thick-appearing capillary loops and spike-like formation under light microscope, IgG and C3 deposits along the capillary wall by IF, and subepithelial electron-dense deposits by ultrastructural evaluation using electronic microscope. HBsAg and/or HBcAg deposits were detected in the same pattern as IgG in all patients, as well as renal PLA2R. Compared with IMN patients, more patients in HBV-MN group showed mesangial electron-deposits (71.4 vs. 15.2%, *P*=0.004), with 3 out of 7 HBV-MN patients accompanied by IgM positive and 2 by IgA positive, indicating secondary membranous nephropathy. There were no other significant differences regarding pathology between the 2 groups (Tables 3 and 4).

**Table 3:** Pathological features of the 7 patients with PLA2R positive HBV associated MN

**Patient**	**Light microscope**			**Immunofluorescence**								**Electronic microscope**	
	Sclerotic glomeruli	Segmental sclerosis	Mesangial proliferation	IgG	IgA	IgM	C3	C1q	C4	HBsAg/HBcAg	PLA2R-Ag	Subepithelial EDs	Mesangial EDs
**1**	0/22	0/22	±	1+	±	+	±	-	-	+/-	+	+	+
**2**	1/40	0/40	±	3+	-	-	2+	-	-	+/-	+	+	+
**3**	0/23	0/23	±	3+	-	+	1+	-	ND	+/-	+	+	±
**4**	0/5	0/5	±	2+	-	-	1+	-	-	+/-	+	+	-
**5**	0/19	1/19	+	4+	+	-	3+	-	-	+/+	+	+	+
**6**	1/2	0/2	_+_	2+	-	-	-	-	-	-/+	+	+	-
**7**	1/17	0/17	±	3+	-	+	2+	-	-	+/+	+	+	+

*HBsAg* Hepatitis B virus surface antigen, *HBcAg* Hepatitis B virus core antigen, *PLA2R-Ag* Phospholipase 2 receptor antigen, *ED* Electron dense deposit, *ND* No data

**Table 4:** Pathological features of PLA2R positive HBV-MN patients and idiopathic MN patients

	**HBV-MN**	**Idiopathic MN**	***P* value**
**Glomerular lesions**			
Sclerotic glomeruli, n (%)	3/7 (42.9)	37/69 (53.6)	0.702
Segmental sclerosis, n (%)	1/7 (14.3)	8/67 (11.9)	1.000
Mesangial proliferation, n (%)	7/7 (100.0)	56/69 (81.2)	0.596
**Immunofluorescence**			
IgG, n (%)	7/7 (100)	69/69 (100)	
C3, n (%)	6/7 (85.7)	65/69 (94.2)	0.392
IgA, n (%)	2/7 (28.6)	8/69 (11.6)	0.228
IgM, n (%)	3/7 (42.9)	12/68 (17.6)	0.138
C1q, n (%)	0/7 (0.0)	7/69 (10.1)	1.000
HBsAg, n (%)	6/7 (85.8)	-	
HBcAg, n (%)	3/7 (42.9)	-	
**Electronic microscopy**			
Subepithelial deposits, n (%)	7/7 (100.0)	46/46 (100.0)	
Mesangial deposits, n (%)	5/7 (71.4)	7/46 (15.2)	0.004

*PLA2R* Phospholipase A2 receptor, *HBV* Hepatitis B virus, *MN* Membranous nephropathy, *HBsAg* Hepatitis B virus surface antigen, *HBcAg* Hepatitis B virus core antigen

Treatment and outcome

The median follow-up for 7 HBV-MN patients was 58 months. They all received entecavir and RAS blockade right after renal biopsy. One patient received immunosuppressive therapy in addition to (two weeks after) entacavir due to severe nephrotic syndrome with acute myocardial infarction and elevated serum creatinine. He firstly received prednisone plus FK506, and change to steroids plus cyclophosphamide at the 11^th^ months because the nephrotic syndrome persisted, and achieved remission 6 months later. Other six patients all achieved remission, including 3 CR and 3 PR after one to three years’ antiviral treatment. The remission rate was 14.3%, 71.4% and 100% at 1, 2 and 3 year respectively. None of 7 patients discontinued antiviral therapy at the last follow-up, and all patients showed stable renal function (Table 1). Compared with IMN group, the prevalence of remission without immunosuppressive therapy of HBV-MN patients was higher (85.7% vs. 43.7%), while the percentage of patients receiving immunosuppresants was lower (14.3% vs. 47.9%) (*P*=0.048) (Table 2).

## Discussion

Since over half of the HBV-MN patients has been found to be PLA2R positive-a feature of idiopathic MN, descriptions of clinical features and prognosis of this entity are lacking. Our study showed the clinicopathological features and outcomes of 7 PLA2R positive HBV-MN patients with complete follow-up data in our center and compared them with PLA2R positive IMN patients.

There is currently no universally accepted diagnostic criteria for HBV-MN. The 2012 Kidney Disease Improving Global Outcomes (KDIGO) guidelines proposed that the diagnosis of HBV mediated glomerulonephritis includes detection of the virus in the blood and the exclusion of other causes of glomerular disease. While some studies used this criteria [10], most researches diagnosed the disease based on more stringent criteria established by a Chinese study which requires the presence of both serum and renal HBV antigens, consistent with the 2021 KDIGO guidelines [9]. The 7 patient in our study were diagnosed according to such criteria. However, attributed to the importance of PLA2R in diagnosis and monitoring of IMN, concern about coincident occurrence of the two diseases was raised. In this study, we showed that though the 7 PLA2R positive HBV-MN patients shared similarities with regard to age, proteinuria severity and renal function with PLA2R positive IMN patients, they also presented some unique clinical features. These patients tended to be female dominant and had more severe hematuria, though there was no statistically significant difference which may be due to small sample size. In terms of pathology, kidney-biopsy specimens of HBV-MN patients showed more mesangial eletron-deposits, a hint of secondary MN. Importantly, we found that patients with PLA2R positive HBV-MN presented favorable outcome after antiviral treatment, showing more remission without immunosuppresants than IMN patients. All above indicated the presence of a secondary form of MN. Detectable PLA2R antigen or antibody in these patients may suggest the autoimmunity of PLA2R triggered by HBV infection.

HBV-MN usually progresses slowly; however, approximately 30% of adult patients was reported to progress to renal failure, and 10% of these patients required renal replacement therapy [5]. The treatment of HBV-MN remains controversial. Antiviral therapy including interferon alpha (IFN-α) and nucleoside analogues was the first line treatment recommended by KDIGO guideline, which is effective in inducing long term remission in patients with HBV-MN [11–14]. However, owing to the poor prognosis, the necessity to use immunosuppressive therapy was raised in an attempt to achieve earlier relief of symptoms and improve prognosis. Prednisone was first used in 1991, showing no help but virus activation [15, 16]. To avoid the exacerbation of liver impairment, combination regimen of antiviral drugs and immunopressants was then introduced. It did improve the proteinuria in HBV-MN patients without altering HBV replication or damaging liver functions [17]. However, only early remission rate were increased compared with antiviral therapy alone [18]. To date, no evidence about the long-term effects of these regimen on renal functions has been published. In this study, patients were all treated with antiviral drugs with only one receiving immunosuppresants. All the patients receiving non-immunosuppressive therapy achieved remission with stable renal function during the follow-up. In contrast to the previous study on HBV-MN, this result indicated a favorable outcome of PLA2R positive HBV-MN. What’s more, this entity showed higher remission rate without immunosuppressive therapy than PLA2R positive IMN patients as the severity of nephrotic syndrome and renal function were similar in the two groups. It’s reasonable for HBV-MN patients to achieve remission of proteinuria after using antiviral drugs, since the deposition of HBeAg and anti-HBe immune complexes detected in the subepithelial region is suggested pivotal to disease pathogenesis [19]. Still, more conservative treatment and longer observation period for HBV-MN patients in our center could partially attributed. Based on our findings, antiviral treatment should be recommended for PLA2R positive HBV-MN patients once the diagnosis established while immunosuppressive therapy would be unnecessary unless impaired renal function or lethal complications associated with severe nephrotic syndrome. Notably, there are one to three years’ antiviral treatment before the 6 patients in our study achieving spontaneous remission, which means a much longer observation period than in the treatment of IMN patients.

Nevertheless, this study was limited by its retrospectively observational analysis, small size and single-center experience. The 7 HBV-MN patients enrolled were with relatively mild involvement as less than half presented with severe nephrotic syndrome. In addition, the lack of PLA2R negative HBV-MN patients and the comparison between PLA2R positive and negative groups limited the investigation on the role of PLA2R in HBV-MN. Thus, a prospective, multi-center, randomized control trial including PLA2R negative HBV-MN patients is needed for further evaluation.

In conclusion, PLA2R positive HBV-MN usually presented with nephrotic syndrome, similar to IMN patients, but much more profound mesangial electronic deposits revealed by renal histopathology. In addition, PLA2R positive HBV-MN patients appears to have more favorable prognosis after antiviral therapy, which supports a secondary form of MN and effectiveness of antiviral therapy on this entity.

## Data Availability

All data generated or analyzed during this study are included in this published article.
